# Evaluation of implant loosening following segmental pedicle screw fixation in adolescent idiopathic scoliosis: a 2 year follow-up with low-dose CT

**DOI:** 10.1186/1748-7161-9-13

**Published:** 2014-08-24

**Authors:** Kasim Abul-Kasim, Acke Ohlin

**Affiliations:** 1 Division of Neuroradiology, Diagnostic Centre for Imaging and Functional Medicine, Faculty of Medicine, Lund University, Skåne University Hospital, Malmö 205 02, Sweden; 2 Department of Orthopaedic Surgery, Faculty of Medicine, Lund University, Skåne University Hospital, Malmö 205 02, Sweden

## Abstract

**Background:**

The long term radiological status of screw fixation following scoliosis surgery with all pedicle screw construct is not previously studied.

**Aim:**

To evaluate the incidence of loosening (implant failure) evaluated with low-dose CT two years following scoliosis surgery.

**Study design:**

Retrospective study.

**Methods:**

81 consecutive patients with adolescent idiopathic scoliosis (AIS), aged 18 ± 3 years at 2 years follow-up (83% were female), subjected for scoliosis corrective surgery with all pedicle screw construct (total of 1666 screws) has been examined with plain radiography and with low dose CT 6 weeks and 2 years postoperatively.

**Results:**

In 26 out of 81 (32%) patients there were signs of loosening of one or more screws, a maximum 3 screws. 47 out of 1666 (2.8%) screws showed evidence of loosening. Preoperative Cobb angle was 56° among patients with loosening compared with 53° among patients with no evidence of loosening (P = 0.288). In males there were signs of loosening in 8 out of 14 (57%) and in females 18 out of 67 (27%), (P = 0.027). Among cases with loosening, 14% had suboptimal screw placement at the first postoperative CT compared with 11% among patients with no evidence of loosening (P = 0.254). One patient with a loosened L4 screw had neurological deficit and subjected for revision of the construct. Out of 26 patients with evidence of loosening, 5 patients reported minor pain or discomfort, 1 patient had a minor proximal junctional kyphosis of about 15° and 3 patients showed evidence of pull-out of 3–5 mm at the upper end of the construct but no clinical complaint. With plain radiography loosening could be observed only in 11 out of 26 cases, 5 were in the lumbar region.

**Conclusions:**

In a consecutive series of 81 cases with AIS who had underwent scoliosis surgery, one third showed, 2 years after the intervention, minor screw loosening. Males were more prone to develop screw loosening. In CT system that enables low-dose protocol, CT is recommended for the evaluation of evidence of screw loosening.

## Introduction

Segmental pedicle screw fixation nowadays is a well established and commonly used fixation technique in the correction and posterior fusion of scoliotic deformities. In our institution, we have developed a CT-technique with extremely low radiation dose, which today is used as a routine examination during the preoperative and postoperative work-up of scoliosis surgery [1-3].

Reports have shown that implant loosening is a well-known complication of this widely used operation technique. The etiology of screw loosening is not well known and believed to be multifactorial. Patient’s age, gender, weight bearing forces and probably muscular forces might contribute to the development of screw loosening. However, the biomechanics of screw loosening need further investigation. The evidence of loosening has been previously studied in patients operated on with pedicle screw fixation for lumbar degenerative diseases [4,5] and showed to vary in incidence depending on type of the screws, operation technique, length of the stabilized segments etc. To our knowledge, there is no report on the long term radiological status of screw fixation among patients with adolescent idiopathic scoliosis (AIS) operated on with segmental pedicle screw fixation. The aim of this study was to evaluate the incidence of loosening (as one of the early signs of implant failure) evaluated with low-dose CT two years following scoliosis surgery. Subsequent aims were to evaluate the degree of correction loss in the short term and to find out if the occurrence of loosening had any impact on the clinical outcome. Furthermore, we aimed to compare the rate of screw loosening detected by CT with that using plain radiography.

## Methods

All patients with AIS operated on with segmental pedicle screw fixation in our University Hospital between February 2006 and December 2010 have been examined with low dose CT 6 weeks and 2 years, respectively following surgery. All CT-examinations were performed on a 16-slice CT-scanner (SOMATOM Sensation 16, Siemens AG, Forchheim, Germany) according to the low-dose protocols. At 6 weeks control the patients were examined with tube voltage of 80 kV, quality reference mAs of 25, computed tomography dose index (CTDI) of 0.54 mGy and dose length product (DLP) of 20 mGycm (average effective dose 0.38 mSv). At 2 years-control the corresponding parameters were 100 kV, 25 mAs, 0.7 mGy and 30 mGycm (average effective dose 0.54 mSv). The effective dose is the tissue weighted sum of radiation taking into account the type of radiation and the nature of each organ or tissue being exposed for radiation. The slightly higher radiation dose at 2-years control was used to acquire better image quality enabling better detection of the evidence of loosening. At the same day the patients underwent low-dose standing plain radiography. The following findings were evaluated by an experienced senior neuroradiologist with specialized profile in the radiology of spinal deformity: (1) Evidence of screw loosening defined as occurrence of radiolucency around pedicle screws [6], (2) evidence of pull-out or change of screw placement status compared with CT at 6 weeks following surgery, (3) coronal Cobb angle on standing radiography to evaluate the loss of correction, and (4) the rate of screw misplacement evaluated according to the grading proposed by Abul-Kasim et al. [2,3] according to the following:

1- Medial cortical perforation of the pedicle (MCP):

Grade 0: Acceptable placement. Screw passes totally within the pedicle medullary canal or with minimal breach of medial pedicular cortex (< ½ of the screw diameter passes medial to medial pedicular cortex).

Grade 1: Partially medialized screw (> ½ of the screw diameter passes medial to medial pedicular cortex).

Grade 2: Totally medialized screw (screw passes totally medial to medial pedicular cortex).

2- Lateral cortical perforation of the pedicle (LCP):

Grade 0: Acceptable placement. Screw passes totally within the pedicle medullary canal or with minimal breach of lateral pedicular cortex (< ½ of the screw diameter passes lateral to lateral pedicular cortex).

Grade 1: Partially lateralized screw (> ½ of the screw diameter passes lateral to lateral pedicular cortex).

Grade 2: Totally lateralized screw (screw passes totally lateral to lateral pedicular cortex).

3- Anterior cortical perforation of the vertebral body (ACP)

Grade 0: Acceptable placement. The screw tip is contained within the vertebral body.

Grade 1: Anterior cortical perforation. The screw tip penetrates the anterior cortex of the corresponding vertebral body. The degree of perforation is reported in mm.

4- Endplate perforation (EPP)

Grade 0: Acceptable placement. The screw tip is contained within the vertebral body.

Grade 1: Endplate perforation. The screw tip penetrates the upper or lower endplate into the adjacent disc space.

5- Foraminal perforation (FP)

Grade 0: Acceptable placement. The screw tip does not penetrate the pedicle border into the overlying or underlying neural foramen.

Grade 1: Foraminal perforation. The screw tip penetrates the pedicle border into the overlying or underlying neural foramen.

A senior spinal surgeon has thoroughly scrutinized the medical records of all patients included searching for the reports of pain, neurological deficit or any other complaint reported at the 2-years visit planned at the same day as the CT and plain radiography.

All operations were performed under general anesthesia with spinal cord monitoring using motor evoked potentials (MEP). The operations were performed according to the technique described by Suk. Self-tapping screws with uniplanar titanium screw head construct were regularly used. Entry points and trajectories for screws were determined by means of metal markers and with aid of fluoroscopy. Screw tracts were prepared by a hand driven drill. The over- contoured rod was then inserted on the concave side of the deformity followed by simple rod rotation and direct vertebral rotation (DVR). Finally, decortication of the posterior element and local bone graft were done to enhance fusion.

The use of low-dose spine CT in the work-up of patients with AIS was approved by the Regional Radiation Protection Committee.

### Statistical analysis

All statistical analyses were performed by means of SPSS (originally; Statistical Package for the Social Sciences) version 21. Data are presented as proportions (%) or as mean ± with standard deviations (SD). Chi square test was performed when studying association between categorical variables whereas Mann–Whitney U test was used when studying association between categorical and continuous variables. The agreement between the occurrence of evidence of loosening on plain radiography and on CT was evaluated by cross tabulation and kappa statistics. Kappa statistics were interpreted according to the method proposed by Landis [7]. Statistical significance was set to a *P* value ≤ 0.05.

## Results

**Figure 1:**
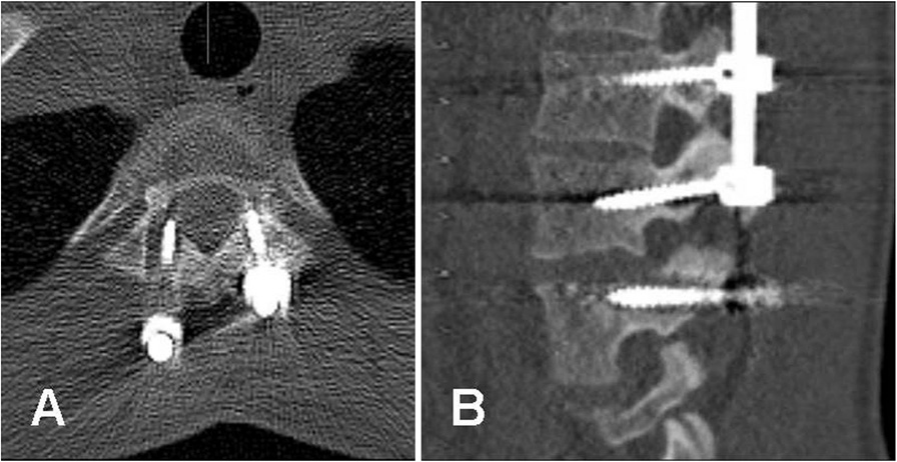
**Evidence of screw loosening.** CT of a 17 years old girl following segmental pedicle screw fixation T3-L3: **(A-B)**. **(A)** axial image showing evidence of loosening around the screw through the right pedicle of T3 and to lesser extent around the screw through the left pedicle. **(B)** sagittal image at the lumbar level showing evidence of loosening around screw through the left pedicle of L3.

A total of 81 patients with AIS operated on with segmental pedicle screw fixation were examined with plain radiography and low dose CT 2 years after surgery (24,6 ± 3 months). Sixty-seven patients were females (83%). The mean age at the 2-years control was 18 ± 3 years (16 years when operated). A total of 1666 screws were evaluated. Signs of loosening of one or more screws (a maximum 3 screws) were found in 26 out of 81 (32%) patients (Figure 1). 47 out of 1666 screws (2.8%) screws showed evidence of loosening by CT. We observed loosened screws at the upper end of the construct in 16 cases, at lower end in 4 and at both ends of the construct in 6 cases. Preoperative Cobb angle was 54 ± 11° for the whole study cohort , 56° ± 11° among patients with evidence of loosening, and 53° ± 11° among patients with no evidence of loosening, p = 0.288, Table 1. The correction rate (the difference between the preoperative and postoperative Cobb angle X 100/preoperative Cobb angle) was 70% in the whole study cohort as well as in both subgroups. Among males there were signs of loosening in 8 out of 14 (57%) compared with 18 out of 67 among females (27%), p = 0.027, Table 2.

**Table 1:** The association between the occurrence of loosening and different continuous variables (Mann–Whitney U test)

	**Whole study cohort**	**Patients with no evidence of loosening**	**Patients with evidence of loosening**	**P-value**
Age (year)	18.5 ± 3	18.6 ± 3	18.5 ± 3	0.878
Preoperative Cobb, major curve (°)	54 ± 11	53 ± 11	56 ± 11	0.288
Postoperative Cobb, major curve (°)	16 ± 7	16 ± 7	16 ± 7	0.804
Correction rate (%)	70 ± 11	70 ± 11	70 ± 12	0.816
Misplacement rate (%)	12 ± 11	11 ± 11	14 ± 11	0.254
Time after surgery	24 ± 3	24 ± 3	24 ± 3	0.671

**Table 2:** The association between the evidence of loosening and different categorical variables

				**Total**	**P-value**
		**No loosening**	**Loosening**		
Gender	Male	6 (43%)	8 (57%)	14	
	Female	49 (73%)	18 (27%)	67	0.027
Pain	No pain	55 (72%)	21 (28%)	76	
	Pain	0 (0%)	5 (100%)	5	0.001
Curve level	Thoracic	41 (69%)	18 (31%)	59	
	Lumbar/thoracolumbar	14 (64%)	8 (36%)	22	0.616

**Figure 2:**
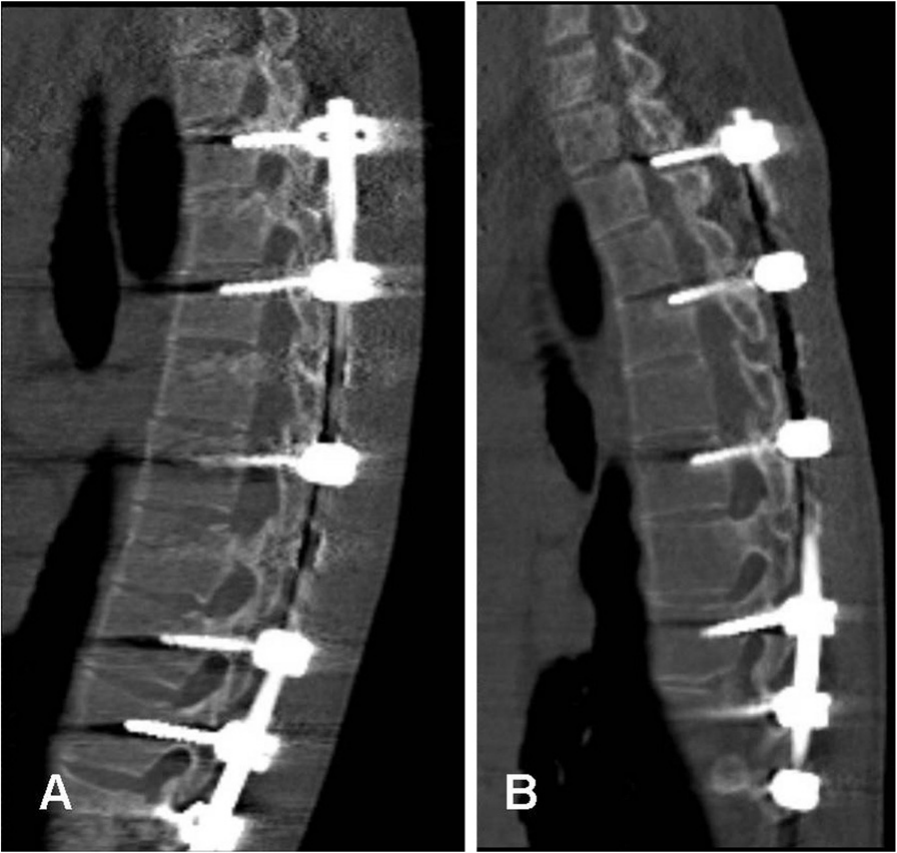
**Evidence of screw loosening and pull out.** CT of a 16 years old boy following segmental pedicle screw fixation T3-L2: sagittal images of one patient, **(A)** six weeks following surgery showing normally placed pedicle screws, and **(B)** two years following surgery showing evidence of pull-out at the level of T3 and T5. The image shows partly evidence of loosening and partly dorsal displacement of the screws and the rod at the upper end of the implant.

**Figure 3:**
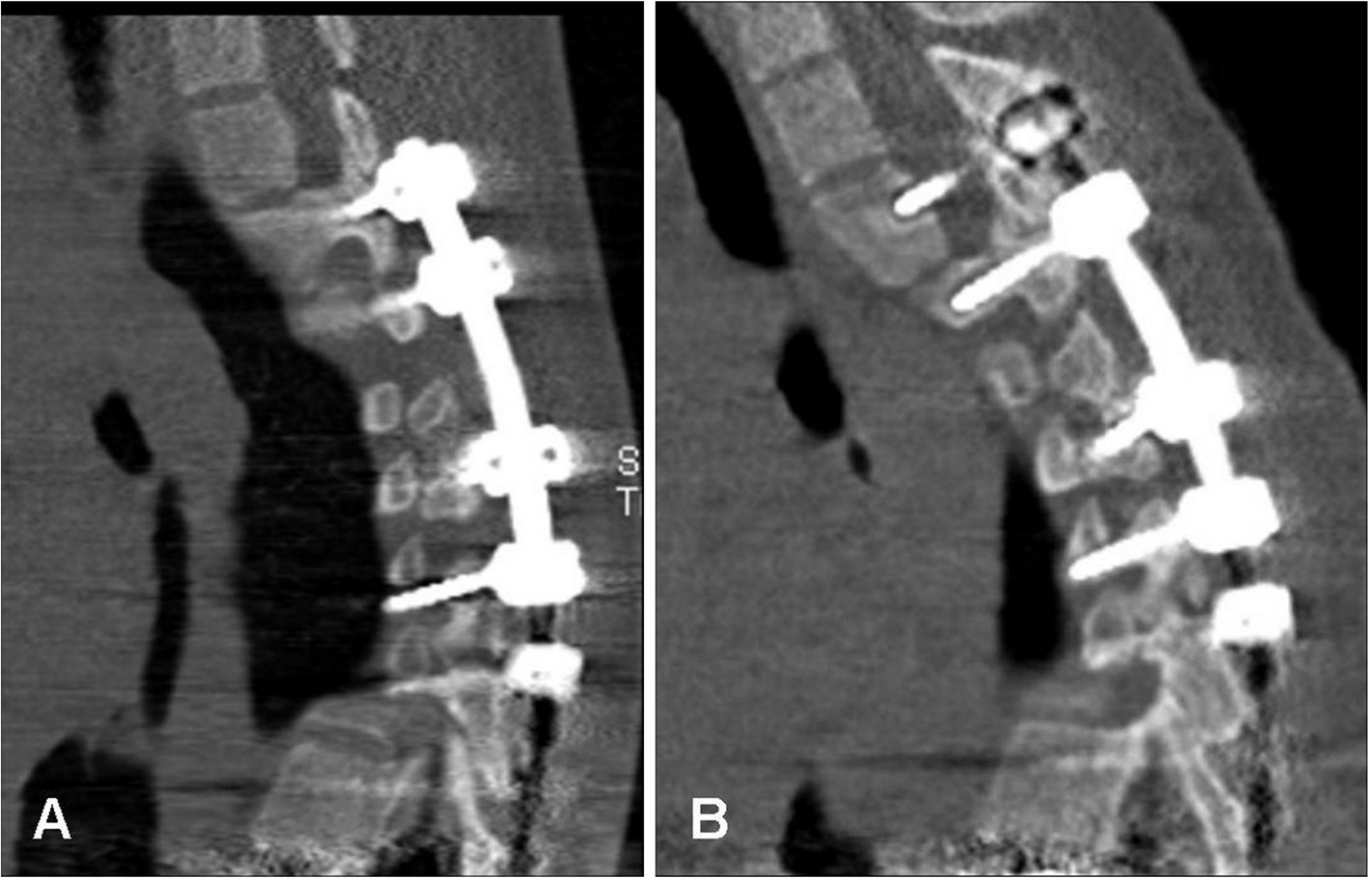
**Evidence of screw loosening and proximal junctional kyphosis.** CT of a 16 years old girl following segmental pedicle screw fixation T2-T12. Sagittal images **(A)** six weeks, and **(B)** two years following surgery of the same patient in Figure 2. Beside evidence of loosening, image **B** shows occurrence of proximal junctional kyphosis above the upper limit of the implant compared with image **A**.

Among patients with evidence of loosening, 14% had suboptimal screw placement in the first postoperative CT compared with 11% among patients with no evidence of loosening, p = 0.254, Table 1. The mean ± SD of the rate of screw misplacement per patient for the whole study cohort was 12 ± 11%. 196 out 1666 (11.8%) screws evaluated showed some degree of misplacement. One patient with a loosened L4 screw had neurological signs and was reoperated with a successful revision. Five out of 26 patients with evidence of loosening reported minor pain or discomfort while none of patients with no evidence of loosening hade pain, p = 0.001, Table 2. Three out of 26 patients with evidence of loosening showed evidence of slight pull-out at maximum of 3 mm at the upper end of the construct but no clinical complaints were reported (Figure 2). Only one patient with evidence of loosening had a minor proximal junctional kyphosis about 15° (Figure 3).

The correction loss of deformity in the coronal plane at 2 years control was 2.4 ± 4.2° for the whole study cohort; 2.5 ± 4° for patients with evidence of loosening and 2.4 ± 4.3° for patients with no evidence of loosening, p = 0.785. The correction loss was higher among patients with residual lumbar/thoracolumbar curves than those with residual thoracic curve (4 ± 5.9° and 1.9 ± 3.3° respectively, p = 0.011). Patients with residual lumbar/thoracolumbar curves hade larger residual Cobb angle than those with residual thoracic curve (23.5 ± 11° and 16.9 ± 5.5° respectively, p < 0.001).

With plain radiography loosening could be observed only in 11 out of 26 cases (42%) with evidence of loosening on CT; 5 were in the lumbar region. This result in kappa value of 0 i.e. poor agreement, Table 3.

**Table 3:** Cross tabulation and kappa statistics for the degree of agreement between the low-dose CT and plain radiography in detecting evidence of loosening

		**Loosening CT**			**Kappa**
		**No**	**Yes**	**Total**	
Loosening plain x-ray	No	55	15	70	
	Yes	0	11	11	
		55	26	81	
					Kappa 0 (P-value 0.001)

The distribution of Lenke types is shown in Table 4. 43% of patients had Lenke type 1. Among them 20% showed evidence of loosening. Evidence of loosening among the other Lenke types-scoliosis is shown in Table 4.

**Table 4:** The occurrence of evidence of loosening and different type of scoliosis classified according to Lenke classification

	**Lenke 1**	**Lenke 2**	**Lenke 3**	**Lenke 4**	**Lenke 5**	**Lenke 6**
No Loosening	28	5	6	3	6	7
Loosening	7 (20%)	3 (38%)	8 (57%)	0	4 (40%)	4 (36%)
Total	35	8	14	3	10	11
P value						0.141

## Discussion

Segmental pedicle screw fixation has the advantage of allowing correction of the 3-dimensional deformity and providing three-column fixation. Implant loosening is a well-known complication of pedicle screw fixation especially in elderly and among patients with osteoporosis. Evidence of implant loosening has been widely studied among patients with lumbar degenerative diseases operated with pedicle screw fixation. Loosening in lumbar surgery 12 months following surgery reported to amount to 7–15% evaluated on plain radiography and 13–26% evaluated on CT [4]. With conventional pedicle screw fixation, the rate of loosening reported to be 19.5% in another study [5]. Röllinghoff et al. showed that 35 out of 64 patients (54%) operated with pedicle screw fixation showed signs of pedicle screw loosening. However, only 7 of these 35 (20%) corresponding to 11% of the whole study cohort complained of back pain [8]. This is the almost the same rate of reported pain among patients with screw loosening in our cohort (5 out of 26, 19% reported pain). Ohlin et al. identified a 40% risk of radiographic evidence for implant loosening or fatigue in a larger patient cohort (n = 163) operated on for different indications such as spinal stenosis, trauma, metastatic spinal disorder, spondylolisthesis, low back pain, ankylosing spondylitis and other miscellaneous conditions [9].

In concordance with Ohtori et al., our study showed great discrepancy between the rate of loosening evaluated on plain radiography and CT [4]. In our study, the degree of agreement between evidence of loosening on plain radiography and CT showed a kappa value of 0, which means that any observed agreement between CT and plain radiography was attributed to chance. CT nowadays is almost a gold standard in the postoperative evaluation of pedicle screw fixation with regard to screw placement [10,11], screw loosening [4] and interbody fusion [12,13] and showed to be more accurate than plain radiography [12,13]. In present study as well as in study reported by Ohtori et al. [4], CT showed higher rate of screw loosening than plain radiography. However, the evidence of screw loosening among patients with scoliosis has, to the best of our knowledge not been studied and reported previously. Furthermore, most of studies reporting implant loosening use plain radiography for their evaluation. Development of our low-dose CT protocols enabled us to perform such evaluation with CT.

Although we did not evaluate bony union in our study, pedicle screw loosening may theoretically negatively influence bone fusion. However, successful bone fusion has previously been shown to be unrelated to the clinical outcome [14]. Wu JC et al. studied the incidence and the long term outcome of dynamic pedicle screw fixation among 126 patients and observed an incidence of loosening of 19.8% [15]. None of the patients with loosening reported any symptom. The clinical outcomes at 3, 12, and 24 months showed no significant differences between the patients with and without screw loosening at all evaluation time points. In a later follow-up, 24% of cases with evidence of screw loosening showed evidence of osseous integration [15].

One of the limitations of this study is that only one reader has done the evaluation of screw loosening without taking into consideration a possible inter-rater variability. However, all evaluations of CT and plain radiography included in this study were performed by a neuroradiologist with a long clinical and scientific experience and with special profile and interest in spinal deformities. In cases where there is any doubt, a consensus was reached at a joined panel with an experienced spinal surgeon.

This study has shown that CT is more sensitive to detect evidence of screw loosening than plain radiography. One of the drawbacks of using CT for evaluating evidence of screw loosening is the possible impact of the artefacts from the metal implant on the accuracy of such evaluation. The increase image noise might have had a negative impact on the detection of the screw loosening. Low radiation dose CT used in our study may induce some halo around the screws resulting in a false positive evidence of screw loosening. On the other hand, image noise might have contributed to miss some cases of true loosening i.e. false negative results. However, we have in previous study reported substantial inter- and intraobserver reliability in the evaluation of screw misplacement on postoperative CT-examinations (6 weeks after surgery) using even a lower radiation dose with subsequently higher image noise [2]. Included in our protocol that is used at 2 years control to evaluate occurrence of screw loosening were partly a slightly higher radiation dose (tube voltage of 100 kV compared with the one used for the initial evaluation of screw misplacement using 80 kV, about 30% higher effective radiation dose), and partly the use of soft tissue algorithm and filter that help reducing the noise from the metal artefacts. As this is the first report on evidence of screw loosening evaluated by CT among patients with AIS operated with posterior pedicle screw fixation, the clinical significance of our finding remain unclear. However, among 26 out of 81 patients with CT-evidence of loosening (one third of patients), one patient had neurological deficit that necessitate surgical intervention, five patients reported pain, three patients showed evidence of pull-out and one patient showed to have PJK. Patients in our study cohort are already enrolled in 5-years clinical and radiological follow-up, which will helpfully bring further knowledge on the clinical significance of the finding of loosening.

Finally, the low-dose CT used in this study showed to be more sensitive than plain radiography in the detection of evidence of screw loosening. Furthermore, the effective radiation dose of low-dose CT is significantly lower than that of plain radiography. In one report, plain radiography of whole spine amounted to 6.36 mSv [16] compared to 0.54 mSv in low-dose CT used in this study. In CT system that enables low-dose protocols, we recommend using low-dose CT in the postoperative evaluation of possible occurrence of screw loosening.

## Conclusion

This study showed that CT is more sensitive than plain radiography in detecting evidence of implant loosening in patients with AIS operated on with segmental pedicle screw fixation. Evidence of loosening on CT occurred in one third of patients, of which one fifth complained of back pain and only one patient had neurological deficit that required implant revision. The loosening was more than twice as common among boys as girls. There was no association between the radiological evidence of loosening and the degree of correction loss two years following surgery. We recommend including low-dose CT in the postoperative work-up searching for evidence of screw loosening as low-dose CT showed to be more sensitive than plain radiography and means exposure to lower radiation dose.

## Competing interests

The authors declare that they have no competing interests.

## Authors’ contributions

KAK has contributed to conception and design of the study, acquisition of data, analysis and interpretation of data, drafting the manuscript and has given his final approval of the version to be published. ACO has contributed to conception and design, acquisition of data, analysis and interpretation of data, and revision of the manuscript critically for important intellectual content, and has given his final approval.
